# DSP-MCF: dual stream pre-training and multi-view consistency fine-tuning for cross-subject EEG emotion recognition

**DOI:** 10.3389/fnhum.2026.1723907

**Published:** 2026-02-18

**Authors:** Jingjing Li, Xinqi Liu, Xia Wu, Ya Wang, Xin Huang

**Affiliations:** 1 School of Artificial Intelligence, Nanyang Normal University, Nanyang, China; 2 Henan Digital Image Big Data Development Innovation Laboratory, School of Artificial Intelligence, Nanyang Normal University, Nanyang, China; 3 School of Information Engineering, Guizhou University of Traditional Chinese Medicine, Guiyang, China

**Keywords:** cross-subject, domain generalization, electroencephalogram (EEG), emotion recognition, multi-view consistency

## Abstract

**Introduction:**

Electroencephalogram (EEG) emotion recognition is attracting increasing attention in the field of brain-computer interface due to its strong objectivity and non-forgery. However, cross-subject emotion recognition is complicated by individual variability, limited availability of EEG data, and interference in certain channels during EEG acquisition.

**Methods:**

We propose a novel synergistic Dual Stream Pre-training and Multi-view Consistency Fine-tuning (DSP-MCF) framework. The DSP-MCF is based on a domain generalization architecture. The framework includes a dual stream pre-training stage, wherein the spatiotemporal encoder-decoder network extracts generalized spatiotemporal representations from masked channels and reconstructs EEG features from incomplete data. Then, a multi-view consistency loss function is proposed during the multi-view consistency fine-tuning. This loss function is essential for aligning the distribution of emotion predictions derived from various perspectives, specifically from actual and masked EEG data.

**Results:**

Experimental results demonstrate that the proposed DSP-MCF framework outperforms state-of-the-art methods in cross-subject EEG emotion recognition tasks. The model achieved an accuracy of 89.76% on the SEED dataset and 77.02% on the SEED-IV dataset.

**Discussion:**

The findings indicate that the DSP-MCF framework effectively addresses individual variability and maintains robust performance even under channel loss. By integrating spatiotemporal reconstruction with multi-view consistency, the model provides a reliable solution for handling incomplete or degraded EEG signals in practical BCI applications.

## 1 Introduction

Emotion is a complex physiological and psychological state that has a significant impact on human physical and mental health, as well as cognitive function, decision making, and social relationships. Consequently, emotion recognition is essential for studying and understanding human emotional expressions (Gao et al., 2019). Electroencephalogram (EEG) technology obtained through brain-computer interface (BCI) is widely employed in healthcare and rehabilitation research because of its authenticity, high accuracy, and capacity to detect the user's emotional state (Fang et al., 2020, 2024; Chen et al., 2021). Advancements in portable, non-invasive BCI sensors and neural network-based emotion identification algorithms have increased interest in EEG-based techniques (Liu R. et al., 2024; Wei et al., 2024). Furthermore, given that the intricate interplay between emotional states and cognitive processes manifests as rapid neural fluctuations, research into EEG emotion recognition has increased dramatically, driven by EEG's exceptional temporal resolution which enables the precise, real-time capture of these dynamic brain activities.

EEG emotion recognition using BCI may be separated into within-subject and cross-subject tasks. Both entail several essential procedures, including brain stimulation, signal acquisition, data processing, feature extraction, and emotion classification. Common features utilized for emotion recognition include Differential Entropy (DE) (Duan et al., 2013) and power spectral density (PSD) (Unde and Shriram, 2014). To further mine discriminative information, tensor-based methods have also been explored to effectively combine multi-scale frequency features (Pei et al., 2021). Initially, the within-subject EEG emotion recognition task was studied using classic machine learning techniques, such as support vector machine (SVM) (Lin et al., 2010). However, the performance was not perfect. The rapid advancement of deep learning has greatly aided the development of within-subject EEG emotion recognition. The combination of convolutional neural network (CNN) and the attention mechanism (Liu et al., 2021) has made significant progress in emotion recognition tasks. Other researchers have been using graph convolutional network (GCN) (Song et al., 2018; Gao et al., 2022) to obtain spatial features by modeling the complicated interaction between EEG channels, because GCN is specially intended to handle irregular and non-euclidean data. At the same time, some studies use CNN and Transformer to synergistically enhance the ability to extract EEG features (Su et al., 2024). The preceding research has made substantial progress in within-subject experiments. However, collecting sufficient labeled EEG data for new individuals is costly and time-consuming, as it requires specialized equipment and controlled environments. This data scarcity problem is further exacerbated by inherent individual differences in EEG responses, which hinder the direct application of within-subject emotion recognition models to new users. To address these limitations, researchers have increasingly focused on cross-subject approaches. For instance, a Dual-Branch Transformer was recently utilized to identify stable spatial-frequency patterns for negative emotion recognition (Pei et al., 2025b).

Due to the considerable variability in individual neural connections and emotional experiences, cross-subject emotion recognition encounters numerous challenges. Models trained on source domain data (i.e., data from multiple individuals) often struggle to generalize effectively to target domain data (i.e., data from new subjects) due to these inherent differences. To address this issue, domain adaptation techniques in transfer learning (Wang and Deng, 2018) have been employed in cross-subject emotion recognition, aiming to extract common emotional features between individuals. Furthermore, unsupervised domain adaptation techniques (Luo and Lu, 2021) have been developed to tackle label-free emotion recognition problems, as labeling EEG data is time consuming and labor intensive. However, these methods often heavily rely on high-quality target domain information. In contrast, domain generalization (Zhou et al., 2022) operates without any access to the target domain during the model training phase, making it particularly suitable for cross-subject EEG emotion recognition tasks.

Cross-subject EEG emotion recognition tasks utilizing domain transfer techniques mainly rely on the temporal or spatial features of EEG signals as input. By adding noise at the time step, the denoising mixed mutual reconstruction (DMMR) technique, which makes use of domain generalization technology, enables the encoder to learn generalized temporal features during the pre-training phase. The fine-tuning step then extracts features related to emotions (Wang et al., 2024). To improve domain invariance representation, a “two-stage prototype contrast domain generalization” (PCDG) network uses a convolutional neural network with residual blocks and convolutional block attention module (CBAM) attention modules to capture spatial information across different channels and electrode positions of EEG signals. This step creates a multi-level prototype contrast mechanism to enhance domain invariance representation (Cai and Pan, 2023). Specifically, manifold-based methods like M3D (Luo et al., 2025) typically perform holistic aggregation by compressing temporal data into static spatial covariance matrices, which inevitably leads to the loss of fine-grained dynamic information. On the other hand, many domain generalization approaches often rely solely on single-domain features, failing to explicitly capture the distinct characteristics of both spatial and temporal dimensions. Although recent studies such as VSGT (Liu C. et al., 2024) have demonstrated the efficacy of independently modeling these dependencies, this strategy remains underexplored in the context of domain generalization. We argue that without explicitly and independently modeling spatial-topological and temporal-dynamic dependencies, it is difficult to effectively align their distinct distribution shifts, thereby hindering the model's ability to handle complex cross-subject variations.

Although cross-subject EEG emotion recognition based on domain generalization technology has made progress in modeling spatiotemporal features, the decoupling of signal spatiotemporal coupling characteristics is still insufficient in the existing methods. It is worth noting that the distribution alignment idea in the pre-training and fine-tuning paradigm provides a new perspective to solve this problem. Inspired by pre-training, minimizing the difference in the distribution of sentiment predictions between the original and masked data can also be considered during fine-tuning. For example, some researchers use the dual-view Kullback-Leibler (DKL) divergence constraint to force the model to generate similar prediction distributions for two different enhanced views of the same image, aiming to align the distribution with the perturbed data (Cao et al., 2024). Other scholars have proposed a self-supervised 3D medical image segmentation framework SwinMM, which employs a masked multi-view encoder in the pre-training stage and introduces KL divergence during fine-tuning to quantify the consistency of segmentation results between different views (Wang et al., 2023). While existing research has explored multi-view quantization in computer vision, current fine-tuning methods in EEG emotion recognition (Wang et al., 2024; Lan et al., 2024) typically rely on either original or masked data in isolation, thereby overlooking the correlation between these two views. It is worth noting that this correlation is particularly critical for EEG signals, the spatiotemporal coupling features are closely related to the multi-channel topological structure, while in real-world scenarios, individual differences or equipment failures often lead to random channel loss.

To simulate the channel loss problem in real-world scenarios, prior research typically employed fixed-threshold or predefined-channel masking strategies. One way to exclude damaged EEG signal fragments is to manually define a variance threshold (Hefron et al., 2018). Another method for simulating channel loss involves adjusting both the number and position of missing channels, ultimately resulting in the retention of five channels (Liu et al., 2022). Although the above strategies are simple and widely used, masking only a set of specific channels will limit the variability and diversity of channel loss, making it difficult to accurately reflect channel damage scenarios in the actual world. In view of this, some scholars have acknowledged the limits of fixed masking strategies, especially the lack of flexibility and the inability to account for the randomness of the real environment, and proposed a dynamic EEG channel masking strategy to address this problem (Lan et al., 2024). Similarly, a pre-training framework based on multi-scale random masked autoencoders (MSMAE), effectively alleviates the data variability and scarcity problems that are prevalent in EEG-based emotion recognition (Li et al., 2022). We adopted a similar approach, but considering that there will not be a large number of damaged channels in the real environment, we adopted a mask strategy of 10%, 20%, and 30%, randomly selecting different mask ratios for each batch, and our strategy will run through the training and testing of the model.

To address the challenges mentioned above in the cross-subject EEG emotion recognition task, we propose a novel framework named Dual Stream Pre-training and Multi-view Consistency Fine-tuning (DSP-MCF). DSP-MCF is based on generalization technology. The approach involves two stages: dual stream pre-training and multi-view consistency fine-tuning, both utilizing just source domain data (EEG signals from current subjects) as input, so ensuring that target domain data (EEG signals from new subjects) remains entirely concealed during model training. Finally, the new subject's emotion recognition accuracy is evaluated under the adverse scenario of partial EEG signal channel loss. To simulate channel loss in actual situations, we introduce a dynamic channel masking approach that increases mask flexibility and variety. During the dual stream pre-training stage, we design an encoder-decoder network framework based on spatiotemporal features, requiring the encoder to deconstruct EEG signals from the dual dimensions of difference and complementarity and learn how to extract generalized spatiotemporal features from masked channels. During the multi-view consistency fine-tuning stage, we discover that there may be changes in the distribution of emotion predictions between original (unmasked) and masked spatiotemporal features. As a result, we propose a constraint mechanism based on the symmetric Kullback-Leibler (KL) divergence. On the one hand, complementary pairs of original masked dual-view data are generated through random mask channels; on the other hand, a bidirectional distribution consistency loss function is designed to construct a symmetric probability distribution alignment space. This approach tries to guarantee that each view has a discriminative feature representation while also dynamically calibrating the emotional state space via implicit probability distribution matching.

The main contributions of this paper are as follows:

1. A novel dual stream pre-training architecture is proposed to capture robust cross-subject invariant spatiotemporal representations from EEG signals with channel loss, optimized through reconstruction loss.
2. A multi-view consistency fine-tuning is proposed, with the primary goal of minimizing the gap between the model's sentiment prediction distributions for original and masked features via KL divergence, hence improving the model's adaptability to incomplete data.
3. A dynamic channel mask strategy is employed to simulate real-world scenarios. The proposed DSP-MCF network obtained an accuracy of 89.76 on the SEED dataset and 77.02 on the SEED-IV dataset, despite the low quality of the data.

## 2 Related work

### 2.1 Cross-subject EEG emotion recognition

Cross-subject EEG emotion recognition aims to broaden BCI application scenarios by overcoming the limitations of within-subject tasks. To capture robust physiological representations, Shen et al. (2022) proposed the CLISA model based on contrastive learning, which integrates spatiotemporal convolutions to achieve robust performance. Similarly, Sartipi et al. (2023) combined spatiotemporal encoding with a recurrent attention network to generate interpretable features. Beyond local features, Guo et al. (2024) introduced the FC-FAN to uncover the intrinsic relationship between functional connectivity patterns and emotional states, while Pei et al. (2025a) addressed the temporal aspect, proposing the weak independence hypothesis to mitigate the mismatch between stimuli and EEG frames. However, while these studies effectively extract meaningful EEG features, they seldom explicitly address the distribution shift between source and target domains.

To mitigate cross-subject variability, domain adaptation techniques have been widely adopted. Representative approaches, such as CiABL (Gong et al., 2024) and DA-CapsNet (Liu S. et al., 2024), minimize subject-specific differences by aligning marginal distributions or employing adversarial mechanisms within capsule networks. Furthermore, multi-task adversarial learning has been leveraged to optimize source subject selection (Qiu et al., 2024). Despite these advancements, these transfer learning approaches typically process spatiotemporal features in a mixed or sigle manner. In contrast, explicitly modeling these dimensions independently offers a more effective pathway to reconstruct original spatiotemporal patterns from corrupted data, thereby enhancing adaptability to target subjects.

### 2.2 Domain generalization

Domain generalization (DG) ensures that the target domain remains completely unseen during training, making it particularly suitable for cross-subject EEG emotion recognition by enhancing adaptability to unseen data. A critical challenge in this field is training on multiple source domains to learn shared features while ensuring robustness to unseen target subjects. To address this, DResNet (Ma et al., 2019) and DGR-ERPS (Li et al., 2024) reduce individual differences by learning shared weights or combining residual networks with time-frequency extraction. More recently, the DMMR framework (Wang et al., 2024) introduced a two-stage strategy integrating self-supervised learning with time-step noise injection to reconstruct generalized features. Given the ability of DG strategies to improve performance on unseen data by strictly excluding the target domain, our approach is also built upon this paradigm.

## 3 Materials and methods

**Figure 1:**
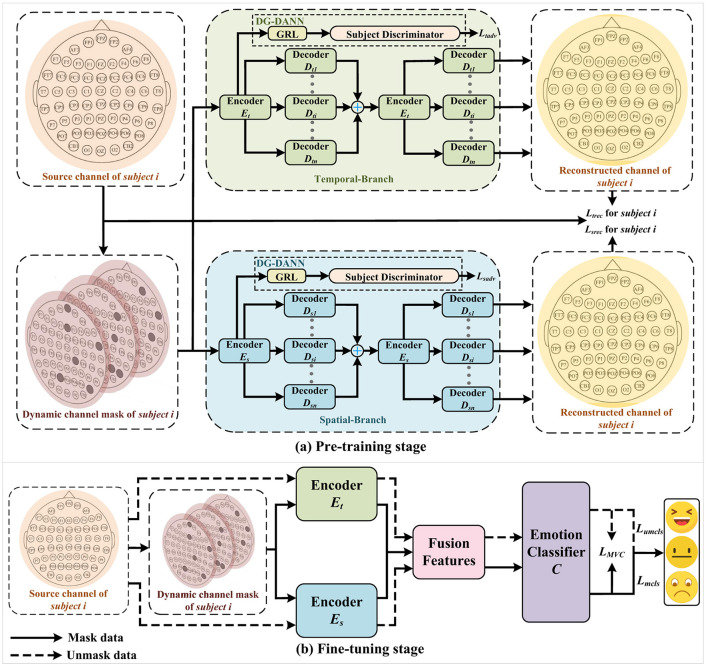
Schematic illustration of the proposed DSP-MCF framework, which comprises two distinct phases. **(a)** Pre-training Stage: This phase aims to learn generalized spatiotemporal representations. It incorporates a Dynamic Channel Masking module (randomly masking 10%–30% of channels) to simulate missing data, followed by a dual-stream autoencoder architecture: a Temporal Branch (LSTM-based) and a Spatial Branch (GCN-based). To eliminate individual variability, a DG-DANN module with a Gradient Reversal Layer (GRL) is applied to both branches, adversarially training the encoder to fool the subject discriminator. **(b)** Fine-tuning Stage: This phase focuses on emotion recognition. The pre-trained encoders process both masked and unmasked views. A learnable parameter μ adaptively fuses the temporal and spatial features. Finally, a Multi-View Consistency Loss (*L*_*MVC*_) minimizes the divergence between the prediction distributions of the masked and unmasked views, enforcing the model to maintain robust performance even under channel loss.

This section delineates the overall architecture of DSP-MCF, as illustrated in Figure 1. To address the prevalent issue of low-quality EEG signals encountered in real-world scenarios, the model employs a dynamic channel mask strategy. DSP-MCF is predicated on a domain generalization technique, which encompasses two distinct stages: pre-training and fine-tuning. The model input comprises data and corresponding labels from *N* source subjects, denoted as $X_{s}={\left\{X_{s}^{i},Y_{s}^{i}\right\}}_{i=1}^{N}$, while data from the target subject is represented by $X_{t}$. Data from these source subjects are utilized to train the model for predicting the emotional states of unseen target subjects. Furthermore, the preprocessing methodology emulates that of Zhao et al. (2021); specifically, features are processed along the temporal axis using overlapping sliding windows, with each window generating segments of *T* time steps. Consequently, the input to the model is formulated as $x=\left(x_{1},x_{2},\ldots ,x_{T}\right)\in {\mathbb{R}}^{T\times C\times B}$, where *C* signifies the number of channels and *B* represents the number of frequency bands.

### 3.1 Dynamic channel mask strategy

Given that a fixed masking ratio may restrict the model's generalizability, particularly in scenarios where channel loss emerges as uncertainty and unpredictability, it is difficult to ensure that a fixed masked channel corresponds to actual channel loss. Our goal is for the encoder to learn domain-invariant features in complex channel degradation scenarios. We address this concern by implementing a dynamic channel mask approach, which increases the flexibility of the model. This approach runs through all processes of the model, aiming to enhance the robustness and generalization capacity of the proposed DSP-MCF.

We implement a dynamic masking strategy by randomly sampling a masking rate from the candidate set {10%, 20%, 30%} with equal probability (*p* = 1/3) for each mini-batch. The selection of these specific masking rates is informed by the observation that in real-world scenarios, typically only a limited number of channels may exhibit compromised data quality. Conversely, high-intensity masking ratios would sever the topological connections among EEG channels, leading to the destruction of intrinsic spatial features. The original feature values of the randomly selected channels designated for masking are subsequently set to zero, representing the masked channel features. The resulting masked channel features, denoted as *x*_*m*_, as shown:


$$\begin{array}{l} x_{m}=x⊙\left(1-M\right) \end{array}$$ (1)

where “⊙” denotes element-wise product, *M* represents a matrix after masking.

### 3.2 Dual stream pre-training

This section provides the implementation details of the proposed dual stream pre-training stage, encompassing both the Temporal-branch and the Spatial-branch.

#### 3.2.1 Temporal-branch

The temporal branch follows the method (Wang et al., 2024), constructing a unified LSTM structure encoder for all subjects and a corresponding multi-decoder system to fully capture the temporal characteristics of the EEG signal. After applying sliding window processing and masking channels to the source data, the mask features *x*_*m*_ after attention weighting are input to the encoder to extract high-dimensional temporal features. Subsequently, the decoder generates temporal reconstruction features in the opposite way to the encoder, and finally applies a linear layer to map the features to the same dimension as the input of this branch, thereby obtaining the output of the *i*-th decoder stage $O_{t1}^{i}\in {\mathbb{R}}^{T\times C\times B}$.

At the same time, in order to make up for the limited EEG data of the source subjects, we draw inspiration from the mixup technology (Zhang et al., 2018) and generate new samples by linearly combining different samples, thereby enriching the training data set. This method adds the outputs of each decoder in the first stage to obtain new mixed subject features. Then, we input the new features into the encoder in the second stage to extract the generalized temporal features again, and use multiple different decoders to reconstruct the features of different subjects in the same category. The output of the *i*-th decoder in the second stage, $O_{t2}^{i}\in {\mathbb{R}}^{T\times C\times B}$, as shown:


$$\begin{array}{l} O_{t2}^{i}=D_{ti}\left(E_{t}\left(\overset{N}{\underset{i=1}{\sum }}O_{t1}^{i}\right)\right),\;i\in \left\{1,2,\ldots ,N\right\} \end{array}$$ (2)

Since the two stages use the same parameters, we use the mean square error (MSE) loss to measure the difference between the reconstructed features and the original features after attention weighting. It aims to minimize the difference between the reconstructed features and the original features to ensure the effectiveness of the reconstructed features. Finally, we get the cumulative reconstruction loss *L*_*trec*_ for *N* instances, as shown:


$$\begin{array}{l} L_{trec}=\overset{N}{\underset{i=1}{\sum }}MSE\left(O_{t2}^{i},r^{i}\right),\;i\in \left\{1,2,\ldots ,N\right\} \end{array}$$ (3)

where, *r*^*i*^∈ℝ^*T*×*C*×*B*^ is the attention-weighted representation of the *i*-th subject, which is the original feature without masked channels and both share the same label.

#### 3.2.2 Spatial-branch

The spatial branch shown in Figure 1a has the same encoder-decoder construction as the temporal branch. However, this structure is based on the GCN framework, which allows for the effective collection of frequent local information between EEG channels and the combination of features from surrounding nodes. The main challenge is to design a suitable adjacency matrix for the EEG channels. Inspired by the works of Salvador et al. (2005) and Zhong et al. (2020), we employ a sparse graph matrix based on the relative spatial placements of the electrodes while ensuring adequate sparsity in the adjacency matrix, which is generated utilizing the spatial distances between various electrodes, as shown:


$$A=\{\begin{array}{ll} 1 & \text{if }A_{ij}\geq 1\\ \frac{\delta }{d_{ij}^{2}} & \text{if }0.1\leq A_{ij}\leq 1\\ 0.1 & \text{if }A_{ij}\leq 0.1 \end{array}$$ (4)

where, *d*_*ij*_ represents the three-dimensional distance between nodes *i* and *j*, and δ is the decay factor. The decay factor is empirically set to δ = 8 based on sensitivity analysis to achieve an optimal balance between local feature aggregation and noise suppression. Furthermore, the adjacency weights *A*_*ij*_ are strictly constrained within the range [0.1, 1]. A lower threshold of 0.1 is applied to prune weak connections and enforce graph sparsity, and the upper bound prevents numerical instability from distance singularities.

**Figure 2:**
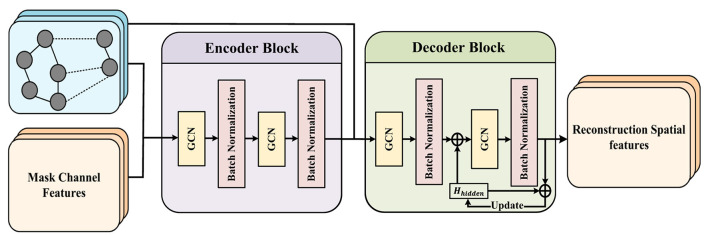
Detailed architecture of the Spatial Branch based on a Graph Convolutional Autoencoder. The process begins by constructing an Adjacency Matrix derived from the 3D physical coordinates of EEG electrodes to represent spatial topology. The Encoder (composed of GCN layers) aggregates local neighborhood information from the input masked features to extract high-level latent spatial representations. Subsequently, the Decoder progressively reconstructs the original spatial features from these latent representations. This mechanism forces the model to capture the intrinsic topological dependencies and spatial integrity of the EEG signals.

Subsequently, we constructed a GCN-based encoder, as shown in the encoder block section of Figure 2, to highlight the distinctive construction of the encoder. The encoder *E*_*s*_ performs classical graph convolution using the masked features *x*_*m*_ and sparse graph matrix *A* as inputs. The propagation of each graph convolution based on relative spatial positions can be expressed as shown:


$$\begin{array}{l} H^{\left(l+1\right)}={\tilde{D}}^{-\frac{1}{2}}A{\tilde{D}}^{-\frac{1}{2}}H^{\left(l\right)}W^{\left(l\right)}+b^{\left(l\right)} \end{array}$$ (5)

where *H*^(*l*+1)^ represents the output of the (*l*+1)_*th*_ GCN layer, *D* is the degree matrix, *W*^(*l*)^ is the trainable weight matrix of the *l*_*th*_ layer, *b*^(*l*)^ is the trainable bias of the *l*_*th*_ layer.

The spatial features gathered by GCN are sent through a standard batch normalization layer to obtain two outputs of the encoder *E*_*s*_, local features and global features. The local features are the input of the decoder, while the global features are the input of the GRL module in Figure 1a. The output *E*_*output*_ of the encoder *E*_*s*_ can be expressed as shown:


$$E_{output}=(BN^{\left(l+1\right)}(H^{\left(l+1\right)}),GlobalPooling(BN^{\left(l+1\right)}(H^{\left(l+1\right)})))$$ (6)

where “,” is the delimiter, the left side represents local features, indicating the features of all channels, and the right side represents extracted through mean pooling to obtain global features. *BN*^(*l*+1)^(·) denotes batch normalization of the *l*_*th*_ layer, and *GlobalPooling*(·) refers the global pooling.

Correspondingly, the specific structure of the multi-decoder $D_{s}={\left\{D_{s}^{i}\right\}}_{i=1}^{n}$ shown in Figure 1a is the decoder block in Figure 2, which receives the output local-feature of the encoder *E*_*s*_ and calculates it through the GCN of the same structure. At the same time, an all-zero hidden state tensor $H_{hidden}^{0}$ is initialized, and the output of the GCN of the first layer is concatenated with $H_{hidden}^{0}$. Then *H*_*hidden*_ is updated according to the output of each layer of GCN. The purpose is to retain the previously aggregated features in the output of each layer and participate in the current calculation. Finally, the reconstructed features decoded by each decoder are obtained by aggregating features layer by layer. The specific work of the decoder is as shown:


$$H^{\left(l+1\right)}=H_{hidden}^{\left(l\right)}+\rho (BN^{\left(l\right)}(L\cdot (x_{local}\cdot W^{\left(l\right)}+b^{\left(l\right)})))$$ (7)

where ρ(·) represents the ReLU activation function, $L={\tilde{D}}^{-\frac{1}{2}}A{\tilde{D}}^{-\frac{1}{2}}$ denotes the Laplacian matrix, *x*_*local*_ refers to the local-feature output of the encoder *E*_*s*_, and *H*_*hidden*_ represents the hidden state tensor.

Based on the output of multiple decoders in the first stage $O_{s1}^{i}\in {\mathbb{R}}^{C\times B}$, the features are combined to obtain new features. These features are then input into the encoder *E*_*s*_ with the same structure and the multi-decoder with the same structure to obtain the reconstructed features $O_{s2}^{i}\in {\mathbb{R}}^{C\times B}$ of the second stage, as shown:


$$O_{s2}^{i}=D_{si}(E_{s}(\sum_{i=1}^{N}O_{s1}^{i})),\;i\in \{1,2,\ldots ,N\}$$ (8)

Finally, the MSE is calculated between the outputs of multiple decoders and the normalized primitive features *S*^*i*^∈ℝ^*C*×*B*^, and the total reconstruction loss *L*_*srec*_ for *N* decoders is computed using the following:


$$\begin{array}{l} L_{\text{srec}}=\overset{N}{\underset{i=1}{\sum }}\text{MSE}\left(O_{s2}^{i},S^{i}\right),\;i\in \left\{1,2,\ldots ,N\right\} \end{array}$$ (9)

#### 3.2.3 DG-DANN module

We employ the DG-DANN method to construct a multi-class subject discriminator (SD). This module operates on an adversarial minimax principle to extract subject-invariant representations. Specifically, the SD is trained to minimize the classification error of the source subject *p*^*i*^, effectively capturing subject-specific attributes. Simultaneously, a gradient reversal layer (GRL) is added before the discriminator, where the gradient is multiplied by a negative factor −λ during backpropagation. This reversal forces the feature encoder to maximize the discriminator's error, effectively stripping away subject-specific noise and “unlearning” domain distinctions. This adversarial mechanism encourages the encoder to generate generalized subject-invariant features. This module is applied to both the temporal and spatial branches, but only in the first stage of each encoder. In this context, *p*^*i*^ represents the ID of the *i*-th subject, and the temporal adversarial losses *L*_*tadv*_ and spatial adversarial losses *L*_*sadv*_ are calculated by the following:


$$\left\{ \begin{array}{l} L_{tadv}=-\lambda p^{i}\log(SD(E_{t}(x_{m}))),\;i\in \{1,2,\ldots ,N\}\\ L_{sadv}=-\lambda p^{i}\log(SD(E_{s}(x_{m}))),\;i\in \{1,2,\ldots ,N\} \end{array}\right.$$ (10)

The temporal adversarial loss and spatial adversarial loss are controlled by balancing the hyperparameters β and γ, respectively. Finally, in the pre-training stage, the spatiotemporal reconstruction loss and the adversarial loss are combined as the total loss, which can be calculated by the following:


$$\begin{array}{l} L_{pre\text{-}training}=L_{trec}+L_{srec}+\beta *L_{tadv}+\gamma *l_{sadv} \end{array}$$ (11)

### 3.3 Multi-view consistency fine-tuning

As depicted in Figure 1b, during the fine-tuning stage, a multi-view consistency loss is introduced to measure the emotion distributions corresponding to masked features *x*_*m*_ and unmasked features *x*_*um*_. These features are extracted from the source domain data *x*_*s*_ by the pre-trained spatiotemporal encoder. This loss aims to align the predictive distributions derived from *x*_*m*_ and *x*_*um*_, encouraging the model to learn invariant features amidst data perturbations. Consequently, this makes the model's outputs from these two perspectives more congruent, and such consistency contributes to enhancing the model's generalization ability in diverse scenarios.

In this process, pre-trained dual encoders are respectively utilized to reconstruct the spatiotemporal features of the masked channels and the spatiotemporal features of the authentic channel data. Building upon this, a learnable weight parameter, μ, is introduced to effectively fuse the extracted spatiotemporal features. To ensure an unbiased prior with equal initial contributions from both streams, μ is initialized to 0.5. The fusion method is calculated using the following:


$$\{\begin{array}{l} F_{fused\_mask}=\mu E_{t}(x_{m})+(1-\mu )E_{s}(x_{m})\\ F_{fused\_unmask}=\mu E_{t}(x_{um})+(1-\mu )E_{s}(x_{um}) \end{array}$$ (12)

Subsequently, supervised learning is performed using ground-truth emotion labels. The masked spatiotemporal fused features and the unmasked spatiotemporal fused features are independently fed into an emotion classifier *C*, yielding the emotion prediction distributions *x*_*m*_*pred*_ and *x*_*um*_*pred*_ for the two respective views. The specific calculations are given by the following:


$$\{\begin{array}{l} x_{m\_pred}=log(C(F_{fused\_mask}))\\ x_{um\_pred}=log(C(F_{fused\_unmask})) \end{array}$$ (13)

Simultaneously, the cross-entropy loss functions, *L*_*mcls*_ and *L*_*umcls*_, are computed for *x*_*m*_*pred*_ and *x*_*um*_*pred*_, against the ground-truth labels *y*^*j*^∈ℝ^*c*×1^, *c* is the number of emotion categories, as shown:


$$\{\begin{array}{l} L_{mcls}=y^{j}x_{m\_pred},\;j\in \{1,2,\ldots ,c\}\\ L_{umcls}=y^{j}x_{um\_pred},\;j\in \{1,2,\ldots ,c\} \end{array}$$ (14)

Subsequently, a multi-view consistency loss is designed. After obtaining the emotion prediction distributions from the two perspectives (i.e., derived from masked and unmasked data), a symmetric KL divergence loss is utilized. We employ this metric because its unbounded nature provides strong, non-vanishing gradients that accelerate convergence, particularly when the views are initially distinct. Furthermore, it enforces strict mutual alignment, compelling the masked branch to recover the sharp, high-confidence decision boundaries of the unmasked view, thereby ensuring robust and decisive classification. This loss aims to minimize the divergence between these two sets of prediction distributions, thereby compelling the model's outputs to maintain consistency across both views. Such an approach not only enhances the model's resilience to data perturbations but also improves its overall robustness and generalization capability. The specific calculation for the multi-view consistency loss *L*_*MVC*_ is as shown:


$$\begin{array}{l} L_{MVC}=D_{KL}(x_{m\_pred}\|softmax(C(F_{fused\_unmask})))\\ \;+D_{KL}(x_{um\_pred}\|softmax(C(F_{fused\_mask}))) \end{array}$$ (15)

where, *F*_*fused*_*unmask*_ and *F*_*fused*_*mask*_ represent the unmask and mask fused spatiotemporal features. In the KL divergence calculation, the term to the left of the “+” sign computes the KL divergence between the log probability distribution of the masked data and the predicted probability distribution of the unmasked data. Conversely, the term to the right of the “+” sign computes the KL divergence between the log probability distribution of the unmasked data and the predicted probability distribution of the masked data.

Finally, for the optimization strategy in the fine-tuning stage, we formulate the total loss function by combining the cross-entropy loss functions, *L*_*mcls*_ (calculated on predictions derived from masked features) and *L*_*umcls*_ (calculated on predictions derived from authentic unmasked features), with the multi-view consistency loss function *L*_*MVC*_. The total loss function for this stage is given by the following:


$$\begin{array}{l} L_{fine\text{\_}tune}=L_{mcls}+L_{umcls}+\theta L_{MVC} \end{array}$$ (16)

## 4 Experiments

In this section, we conduct extensive experiments on two datasets, SEED and SEED-IV, to verify the effectiveness of our proposed DSP-MCF method.

### 4.1 Datesets and evaluations

To evaluate the performance of the DSP-MCF model, we conduct assessments on two public EEG emotion datasets: SEED (Zheng et al., 2017) and SEED-IV (Zheng et al., 2018).

**SEED** is a public dataset for EEG emotion recognition collected by Shanghai Jiao Tong University that uses carefully selected movie clips (15 emotional clips from movies, each clip about 4 min) to elicit three different emotional states from the subjects: positive, neutral, and negative. It contains data from 15 healthy subjects (7 males and 8 females, aged 23.27 ± 2.37, all right-handed with normal or corrected-to-normal vision), each of whom participated in 3 independent experimental sessions. All participants were native speakers matching the language of the video stimuli. They underwent pre-experiment instructions and familiarization sessions to ensure natural engagement. During the experiment, the EEG signals of the subjects were collected using a 62-channel ESI NeuroScan system with a sampling rate of 1,000 Hz. To minimize physiological interference (e.g., EOG and EMG artifacts) and ensure data quality, we utilized the standard preprocessed Differential Entropy (DE) features. These data had undergone rigorous preprocessing, including downsampling to 200 Hz, bandpass filtering (1–75 Hz), and smoothing using the Linear Dynamic System (LDS) method.

**SEED-IV** follows a similar experimental paradigm to the SEED dataset, also involving 15 distinct subjects (7 males and 8 females, aged 20–24) who met the same inclusion criteria as the SEED cohort. Each subject performed three rounds of experiments, each containing 24 trials, and collected EEG signals as well as eye movement data using emotional video elicitation. The physiological data were collected by a 62-channel ESI NeuroScan system and SMI eye-tracking glasses. Consistent with SEED, standard preprocessed DE features were employed to exclude artifacts. The core difference lies in the emotion labeling scheme, where SEED-IV classifies emotions into four separate categories: neutral, sad, fearful, and happy.

**Evaluation**: To achieve cross-subject evaluation, we use the leave-one-subject-out cross-validation approach, calculating the average (avg.) and standard deviation (std.) across all subjects, where one subject is designated as the test set while the remaining subjects formed the training set. This process is repeated to ensure that each subject served as the target test subject once.

### 4.2 Implementation details

In the experiments, the EEG signals are initially filtered and then further divided into five frequency bands: δ: 1–3 Hz, θ: 4–7 Hz, α: 8–13 Hz, β: 14–30 Hz, and γ: 31–50 Hz. The DE features of EEG signals are extracted from these frequency bands to serve as input. DE features are selected for their superior ability to capture rich sentiment-related information in high-frequency bands compared to other metrics (e.g., PSD). This choice also ensures a strictly fair comparison with state-of-the-art baselines. we utilize the first session from both datasets as the experimental data, following the approach of DMMR (Wang et al., 2024). The input feature dimensions for both datasets are maintained uniformly, with the hidden layer sizes of the temporal and spatial encoders set as 64. The hyperparameter α controlling the reconstruction loss in the spatial decoder is set to 0.5 for the SEED dataset and 0.1 for the SEED-IV dataset. The balance hyperparameters β and γ for the adversarial loss in both the temporal and spatial branches are set to 0.05. We utilize the Adam optimizer with a weight decay of 5e-4. The learning rate is set to 1e-3, aligning with the standard settings of the aforementioned baselines to ensure strict fairness, thereby attributing performance gains to the proposed architecture rather than hyperparameter tuning. Considering the data sizes of SEED and SEED-IV, both the time steps *T* are set to 30 and 10, with batch sizes of 512 and 256, respectively. To ensure reproducibility, the random seed is fixed to 3. We adopt a two-stage training strategy: 120 pre-training and 200 fine-tuning epochs for SEED, whereas SEED-IV requires 200 pre-training epochs followed by 200 fine-tuning epochs. Meanwhile, a “save-best” strategy is employed to retain the model with the highest validation accuracy. All experiments are implemented using Pytorch on the NVIDIA A40 GPUs. For practical deployment, the model is streamlined by retaining only the encoders and classifier while discarding the computationally heavy decoders. Theoretically, the temporal complexity scales linearly with time steps *T*, while the spatial complexity is dominated by the matrix multiplication *O*(*C*^2^). Under this hardware setting, the proposed model achieves an average inference latency of approximately 1.24 ms per sample (with a batch size of 1), fully satisfying the requirements for real-time BCI applications.

### 4.3 Cross-subject emotion recognition results

To validate the efficacy of DSP-MCF in cross-subject emotion recognition, its performance was benchmarked against established baseline models, also evaluated under cross-subject tasks. The exact outcomes are detailed in Table 1. For the comparison analysis, various state-of-the-art deep learning architectures were chosen. These include the graph-convolution-based model DGCNN (Song et al., 2018) and the semi-supervised graph contrastive learning approach DS-AGC (Ye et al., 2024). Furthermore, the unsupervised domain adaptation model BiHDM (Li et al., 2020), DA-CapsNet (Liu S. et al., 2024) (which integrates domain adaptation with capsule networks), and the dual filtration subdomain adaptation network (DFSAN) (She et al., 2025), which is a fine-grained subdomain adaptation network designed to mitigate negative transfer in domain adaptation, were incorporated. Both DResNet and DG-DANN (Ma et al., 2019) employ joint learning domain generalization approaches. PPDA (Zhao et al., 2021) uses a minimal amount of target domain data to fine-tune a pre-trained model. The parallel temporal–spatial-frequency neural network (PTSFNN) (He et al., 2024) is a shallow design capable of processing the temporal, frequency, and spatial domain features of EEG signals. The DMMR (Wang et al., 2024) model adds noise by temporal perturbation and uses pre-training and fine-tuning to address suboptimal model generalization caused by individual variability. Additionally, a method leveraging multi-source structural deep clustering (Chen et al., 2025) to discover intrinsic structural knowledge inside the target domain, regularized by source label distributions, is considered. Distinct from these approaches, DSP-MCF employs a more randomized masked channel strategy to simulate head channel degradation in actual environments. It proposes a dual stream architecture for the pre-training phase to generate a spatiotemporal encoder capable of extracting generalizable features. In the fine-tuning phase, multi-view consistency is used to quantify the emotion prediction distribution between masked and unmasked EEG data. Finally, the use of incomplete EEG data during the testing phase serves to enhance the model's robustness and generalization capabilities.

**Table 1:** Performance comparison of the proposed DSP-MCF with baselines (avg/std%).

**Method**	**SEED**	**SEED-IV**
DGCNN (Song et al., 2018)	79.95/9.02	-/-
DG-DANN (Ma et al., 2019)	84.30/8.32	-/-
DResNet (Ma et al., 2019)	85.30/7.97	-/-
BiHDM (Li et al., 2020)	85.40/7.53	69.03/8.66
PPDA (Zhao et al., 2021)	86.70/7.10	-/-
DA-CapsNet (Liu S. et al., 2024)	84.63/9.09	-/-
DS-AGC (Ye et al., 2024)	87.37/6.19	66.00/7.93
PTSFNN (He et al., 2024)	87.63/5.44	74.96/8.23
DMMR (Wang et al., 2024)	88.27/5.62	72.70/8.01
DFSAN (She et al., 2025)	88.68/6.08	67.61/13.27
SDC (Chen et al., 2025)	88.20/9.69	71.49/13.58
DSP-MCF (Ours)	89.76/5.60^*^	77.02/8.04^*^

The symbol “-/-” indicates unreported in the original paper, and “^*^” denotes statistical significance (*p* < 0.05).

The efficiency of DSP-MCF is further demonstrated by comparing the cross-subject experimental findings from the two datasets. On the SEED dataset, our proposed DSP-MCF obtained an accuracy of 89.76 with a minimal standard deviation of 5.60, outperforming most current approaches (see Table 1). It should be noted that the performance of DSP-MCF on this dataset is 1.56 higher than the latest method Structural Deep Clustering, and 1.49 higher than the baseline model DMMR. Crucially, a paired t-test against the DMMR baseline confirms statistical significance with *p* = 0.0023. As can be seen from Table 1, on the SEED-IV dataset, DSP-MCF achieved an accuracy of 77.02 with a minimum standard deviation of 8.04, which is 9.41 and 5.53 higher than the latest methods DFSAN and Structural Deep Clustering, respectively, and 4.32 higher than the baseline model DMMR. This improvement is also statistically significant (*p* = 0.0177), which shows that the performance of the DSP-MCF model is superior. Overall, experiments on two datasets show that DSP-MCF can work effectively even when processing low-quality EEG signals by obtaining a spatiotemporal encoder with generalization ability in the pre-training stage and aligning the emotion distribution consistency of masked and unmasked EEG signals in the fine-tuning stage.

### 4.4 Single subject results

**Figure 3:**
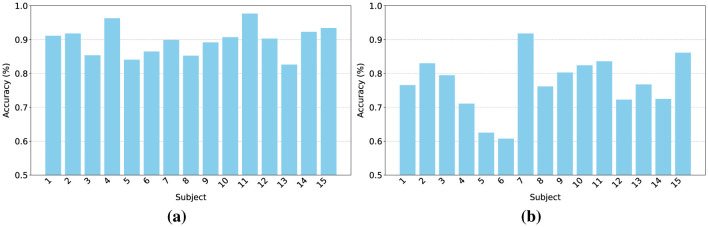
Experimental results of each subject: **(a)** SEED dataset and **(b)** SEED-IV dataset (Accuracy %).

To further validate the effectiveness of our proposed DSP-MCF method in cross-subject emotion recognition tasks, we conducted individual subject emotion recognition accuracy experiments on each of the 15 subjects in the SEED and SEED-IV datasets, respectively. As shown in Figure 3a, the emotion classification results for each subject are presented. It can be observed that on the SEED dataset, due to its inherent three-category emotion classification, the results for each subject exhibit a significant advantage, with all accuracies maintained above 80. The average accuracy for the cross-subject task reached 89.76. Meanwhile, Figure 3b displays the four category emotion classification results for each subject in the SEED-IV dataset. Because it expands upon the SEED dataset to four emotion categories, its performance in the cross-subject task is not as high as that on the SEED dataset; nevertheless, many subjects still achieved outstanding results. Ultimately, the average accuracy on this dataset was 77.02, which is also an excellent result for a cross-subject task. The results from both datasets demonstrate the performance advantages of DSP-MCF.

### 4.5 Ablation study

As shown in Table 2, the ablation experiment results of our proposed method are presented. In order to better analyze the proposed dual-branch spatiotemporal encoder-decoder pre-training network and the fine-tuning network for multi-view consistency measurement of sentiment prediction distribution. Three ablation experiments were designed based on DSP-MCF to verify the effectiveness of its various components. First, the “w/o hidden” in the experiment refers to removing the hidden state update layer in the GCN decoder module, which will make the output obtained in the previous step not used during decoding. From the results in the Table 2, it can be seen that it has dropped by 2.06 on the SEED dataset and by 0.73 in SEED-IV, which can prove that this part has played a certain role. In addition, “w/o Spatial-Branch” means removing the entire spatial branch module and simplifying the original dual-branch structure into a single-branch model, that is, the complementary spatial features are missing. The accuracy of the result on the SEED dataset drops by 2.77, and the accuracy on the SEED-IV dataset drops by 5.01, indicating that the spatial branch can significantly improve the performance of the model. Similarly, the “w/o Temporal-Branch” variant removes the LSTM-based temporal module. This results in a performance drop of 3.55 on SEED and 2.67 on SEED-IV. Comparing the two branches reveals that while the temporal branch is slightly more critical for basic emotion differentiation on SEED, the spatial branch plays a dominant role in the fine-grained tasks of SEED-IV. The optimal performance is achieved only when both branches function synergistically. “w/o *L*_*MVC*_” means removing the calculation of the multi-view consistency loss in the fine-tuning stage. This loss is used to maintain the consistency of the masked and unmasked EEG emotion prediction distributions. After its removal, the accuracy of the result on the SEED dataset drops by 4.35, and the accuracy on the SEED-IV dataset drops by 5.84, fully demonstrating the importance of this loss to the fine-tuning part and its great impact on the overall model effect. The effect of the ablation experiment greatly demonstrates the positive utility of the proposed components in the overall model.

**Table 2:** Effectiveness of each component in our DSP-MCF approach (avg/std%).

**Method**	**SEED**	**SEED-IV**
DSP-MCF	89.76/5.60	77.02/8.04
w/o hidden	87.70/5.22	76.29/10.32
w/o Spatial-branch	86.99/5.59	72.01/7.13
w/o Temporal-Branch	86.21/6.01	74.35/7.89
w/o *L*_*MVC*_	85.41/7.19	71.18/8.83

### 4.6 Impact of masking strategies and robustness analysis

To rigorously validate the effectiveness of the proposed masking strategy, we conducted a comprehensive comparative analysis across different masking modes and intensities, with results summarized in Table 3. First, examining fixed channel corruption ranging from 10% to 60% reveals a clear inverse correlation between the masking ratio and model performance. While mild corruption (10%–30%) yields relatively stable results, high-intensity masking (40%–60%) leads to degradation in accuracy (e.g., SEED drops from 87.90% at 10% to 80.79% at 60%). We attribute this decline to the fact that masking nearly half of the electrodes destroys the global graph topology, severing the critical spatial connections required for effective GCN inference.

**Table 3:** Performance comparison of different masking strategies on SEED and SEED-IV datasets (avg/std%).

**Method**	**SEED**	**SEED-IV**
Fixed mask 10%	87.90/7.49	74.61/8.95
Fixed mask 20%	87.61/8.08	74.36/9.49
Fixed mask 30%	86.06/9.37	72.11/9.89
Fixed mask 40%	84.64/7.05	69.87/9.95
Fixed mask 50%	83.60/7.78	66.45/10.21
Fixed mask 60%	80.79/8.80	65.34/10.57
**Dynamic mask (10%, 20%, 30%)**	**89.76/5.60**	**77.02/8.04**
Dynamic mask (40%, 50%, 60%)	86.58/6.08	70.36/9.37

Bold values indicate the experimental results of our method in selecting the optimal mask ratio.

Crucially, our proposed DSP-MCF (Dynamic 10%, 20%, 30%) consistently surpasses all individual fixed masking baselines within the same range. This empirical evidence validates that fixed masking restricts the model to static noise patterns, potentially leading to overfitting to specific corruption levels. In contrast, the dynamic strategy functions as a form of continuous data augmentation. By randomly sampling ratios during training, the model is forced to learn robust topological correlations that are invariant to the degree of signal loss, rather than relying on fixed reconstruction shortcuts.

To further justify our selection of the 10%–30% range, we compared it against a “High-Intensity” dynamic set (40%, 50%, 60%). The results reveal that the high-intensity set suffers from the same information loss problem observed in high fixed rates, leading to lower accuracy (SEED: 86.58%; SEED-IV: 70.36%). This aligns with established data quality protocols; for instance, the widely adopted PREP pipeline (Bigdely-Shamlo et al., 2015) typically flags datasets with >25% bad channels as unreliable for interpolation. Therefore, the 10%–30% interval represents the optimal trade-off, effectively simulating realistic sensor failures to train robust features without causing catastrophic topological destruction. Notably, even under the high-intensity dynamic setting, our model outperforms the fixed 60% baseline, demonstrating strong resilience against severe sensor failures.

### 4.7 Performance analysis of emotion categories

**Figure 4:**
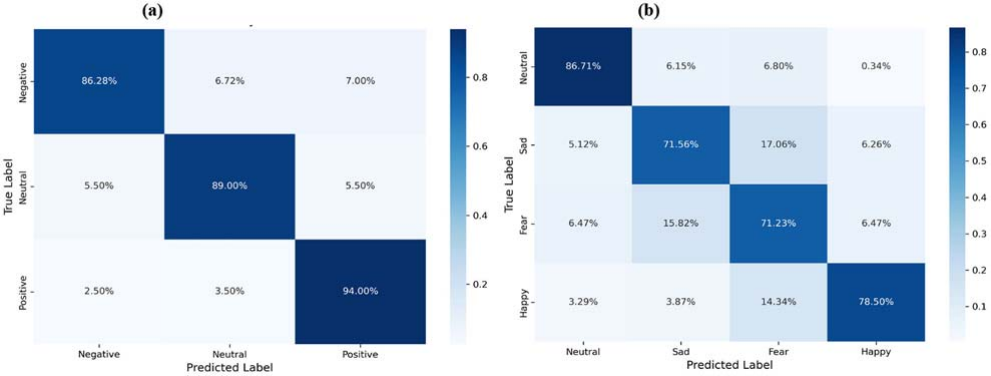
Confusion matrices of emotion recognition results on the SEED dataset (left) and the SEED-IV dataset (right). **(a)** DCP-MCF on SEED. **(b)** DCP-MCF on SEED-IV.

Figure 4 illustrates the confusion matrices to analyze category-specific adaptability. On SEED (Figure 4a), the model exhibits high efficacy, particularly for “Positive” emotion (94.00%), indicating distinct topological patterns. On SEED-IV (Figure 4b), the performance variance provides insight into fine-grained recognition challenges. The primary difficulty stems from high topological ambiguity between intra-valence categories: “Sad” and “Fear” exhibit significant mutual misclassification (approx. 16%–17%) due to overlapping neural activation in the negative valence quadrant. Despite this intrinsic difficulty, DSP-MCF maintains robust accuracy (>71%) for both categories, demonstrating effective discriminative power even under subtle emotional differences.

### 4.8 Visualization for feature distribution

**Figure 5:**
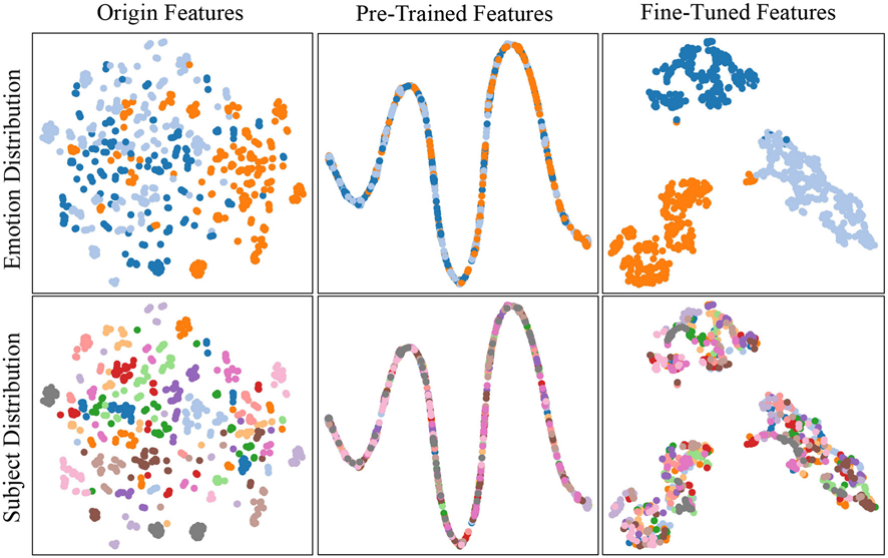
T-SNE visualization of feature distributions for emotion (**top** row) and subject (**bottom** row) across raw, pre-trained, and fine-tuned phases (left to right). The **top** row shows emotion categories (Orange: Positive, Light Blue: Neutral, Dark Blue: Negative) gradually forming distinct clusters. The **bottom** row demonstrates subject invariance, where the target subject (Red) shifts from isolated clusters to a distribution closely aligned with source subjects, validating the removal of individual variability.

The T-SNE algorithm is employed to validate the performance of our model in extracting subject-invariant features, illustrating the boundaries of emotion categories, and demonstrating its discriminative ability. We randomly select 50 samples from each source subject in the SEED dataset for processing and visualize the distribution of the original features in terms of subject and emotion category. Additionally, we present the visualization results during the pre-training and fine-tuning phases. Figure 5 displays the results, with each subject denoted by a distinct hue, and the target subject highlighted in red. The three emotional categories positive, neutral, and negative are represented by orange, light blue and dark blue, respectively.

As observed from Figure 5, the distribution of the original individual features is relatively scattered, both in terms of subject and emotion categories. Since the pre-training phase of our model focuses on extracting shared features among subjects and the fine-tuning phase primarily emphasizes the subdivision of emotion, the emotional distribution in the pre-training phase appears to be overlapping. During the fine-tuning phase, the emotion distribution becomes divided into three categories, with a few misclassified instances remaining. These results show that the pre-training phase captures generalizable features, while the fine-tuning phase refines the emotion boundaries. A close study of the target subject distribution shows that there is a lot of overlap with the source subjects. This demonstrates that DSP-MCF did an effective job of aligning the source and unobserved target domains. Furthermore, because the target domain contains masked channels, these findings confirm that DSP-MCF successfully reconstructs the compromised channels.

## 5 Conclusion

In this paper, a novel DSP-MCF model based on domain generalization technology is proposed to deal with significant individual emotion differences in cross-subject tasks as well as channel loss in real-world contexts. First, in the dual stream pre-training stage, the model uses the spatiotemporal encoder-decoder reconstruction network in conjunction with adversarial training to "force" the spatiotemporal encoder to learn to extract more generalized features from masked EEG features. Then, in the multi-view consistency fine-tuning stage, the pre-trained spatiotemporal encoder is used to extract spatiotemporal features, which are subsequently fused using trainable parameters. At the same time, we also propose a multi-view consistency loss function to measure the consistency of the emotion distribution between masked and unmasked features during the fine-tuning. The model ensures that the target domain data remains fully unseen throughout training, demonstrating its ability to handle cross-subject tasks. To better deal with the channel loss in real BCI scenarios, our model masks the channel of the target test data during inference, ensuring that the model has good generalization ability and strong robustness even in the case of new and unseen damaged channels. Finally, DSP-MCF achieved an excellent accuracy of 89.76 on the SEED dataset and 77.02 on the SEED-IV dataset. However, we explicitly acknowledge that the current study is limited to cross-subject scenarios within a single acquisition system; consequently, cross-device generalization across heterogeneous datasets remains a critical direction for future investigation. Future research can explore more advanced domain generalization techniques based on the target domain's ability to be completely invisible during model training, as well as methods for adaptive mask channels and improving the generalization ability of mask channel features in cross-subject tasks. Furthermore, DSP-MCF may be developed to do real-time EEG processing and integrate it with multimodal data (such as images, text, and other physiological signals) to increase the accuracy of emotion recognition tasks. Overall, our method shows excellent performance in cross-subject EEG emotion recognition tasks.

## Data Availability

Publicly available datasets were analyzed in this study. This data can be found here: SEED: https://bcmi.sjtu.edu.cn/home/seed/seed.html; SEED-IV: https://bcmi.sjtu.edu.cn/home/seed/seed-iv.html.
