# Supplementary material for: DSP-MCF: dual stream pre-training and multi-view consistency fine-tuning for cross-subject EEG emotion recognition
Source: Front Hum Neurosci. 2026 Feb 18;20:1723907. doi: 10.3389/fnhum.2026.1723907 (PMC12957252; doi:10.3389/fnhum.2026.1723907)
Supplement: Supplementary file 1 [file Data_Sheet_1.pdf]

Supplementary Material

1 PARAMETER SENSITIVITY ANALYSIS

To rigorously justify the hyperparameter selection in the DSP-MCF framework, we conducted independent sensitivity analyses for the temporal adversarial weight (β), the spatial adversarial weight (γ), the spatial reconstruction weight (α), the multi-view consistency loss weight (θ) and the decay factor in adjacency matrix construction (δ).

1.1 Adversarial weights (β and γ)

The hyperparameters β and γ control the intensity of the gradient reversal layer (GRL) in the temporal and spatial branches, respectively. These weights are critical for balancing the trade-off between learning discriminative emotion features and achieving subject invariance. We evaluated these parameters within the range of $[0.01, 0.09]$. As illustrated in Figure S1, the classification performance peaks at 0.05 for both β and γ across both datasets. When the weights are too small (< 0.02), the adversarial penalty is insufficient, causing the encoder to retain subject-specific noise. Conversely, when the weights are excessively large (> 0.08), the adversarial loss overwhelms the primary emotion classification loss. This "over-correction" forces the model to discard discriminative emotional semantics in favor of domain invariance, leading to a decline in accuracy. Therefore, $\beta = \gamma = 0.05$ is selected to achieve the optimal equilibrium.

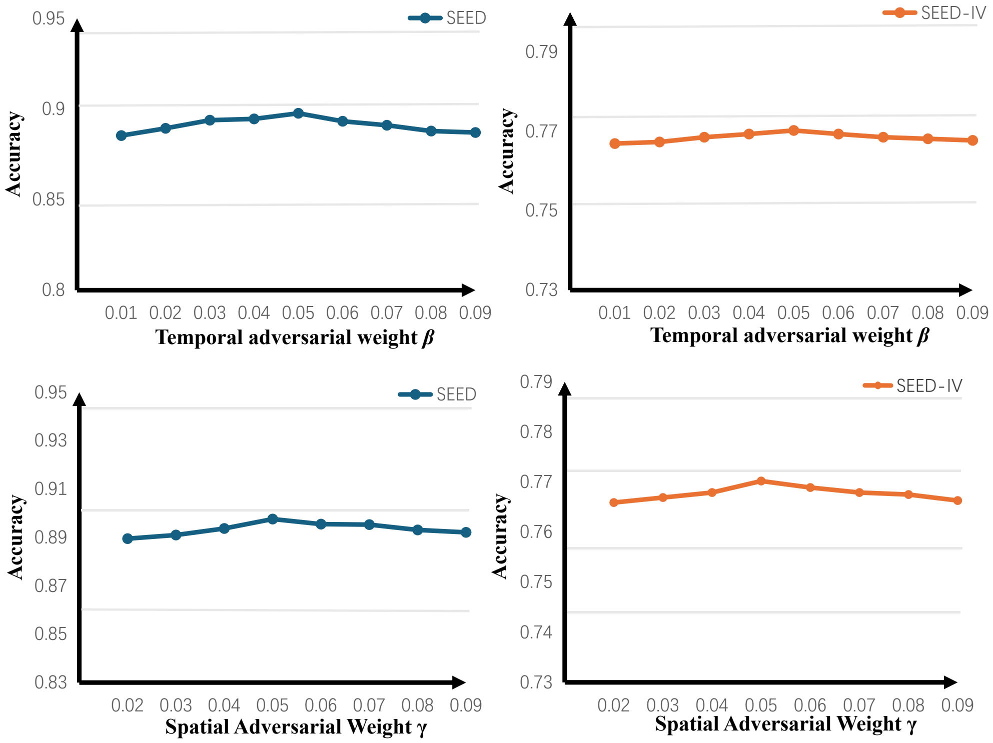

Figure S1. Sensitivity analysis of the adversarial weights on the SEED and SEED-IV datasets. The top row shows the impact of the temporal adversarial weight β, and the bottom row shows the impact of the spatial adversarial weight γ.

1.2 Spatial reconstruction weight (α)

The coefficient α governs the contribution of the spatial decoder, ensuring the model preserves the topological integrity of EEG signals. We performed a search over the range of $[0.2, 0.9]$ for the SEED dataset and $[0.04, 0.18]$ for the SEED-IV dataset. As shown in Figure S2, the optimal values were identified as $\alpha = 0.5$ for SEED and $\alpha = 0.1$ for SEED-IV. The discrepancy in optimal α values is attributed to the inherent data complexity. The SEED dataset exhibits relatively distinct global spatial patterns, benefitting from a higher reconstruction weight (0.5) to capture topological dependencies. In contrast, SEED-IV involves fine-grained emotion discrimination with higher topological ambiguity. A larger α on SEED-IV forces the model to overfit to noisy spatial correlations, potentially causing negative transfer. Consequently, a lower weight (0.1) is adopted for SEED-IV to prioritize discriminative feature learning.

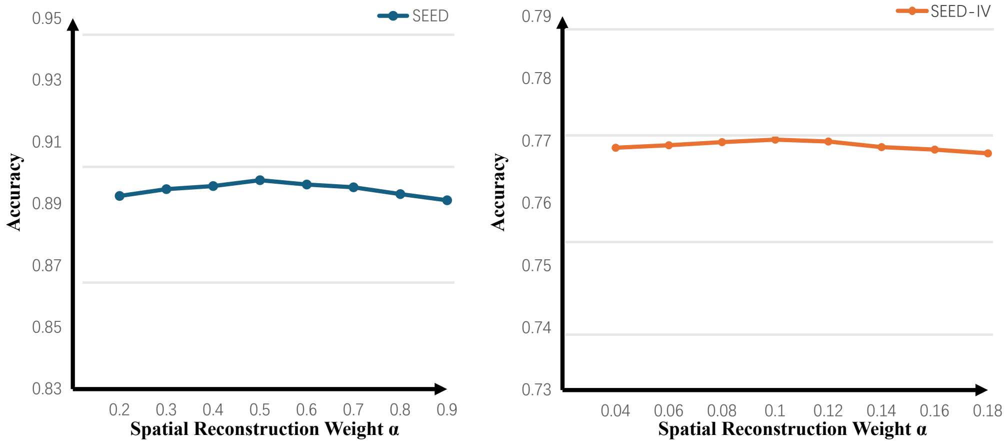

Figure S2. Sensitivity analysis of the spatial reconstruction weight α on the SEED (left) and SEED-IV (right) datasets.

1.3 Multi-view consistency loss weight (θ)

In the fine-tuning phase, the hyperparameter θ serves as a balance coefficient for the Multi-View Consistency Loss (L_{MVC}), regulating the intensity of the alignment between masked and unmasked emotion prediction distributions. As illustrated in Figure S3, the classification accuracy on both SEED and SEED-IV datasets initially increases and reaches a peak at $\theta = 0.5$, after which it declines. This trend suggests a smaller θ (< 0.4) provides insufficient constraint to enforce effective distribution consistency, while a larger θ (> 0.6) introduces excessive regularization that interferes with the primary classification task. Consequently, θ is set to 0.5 to achieve the optimal trade-off.

1.4 Decay factor in adjacency matrix construction (δ)

The decay factor δ, used in the adjacency matrix construction (Equation 4 in the main manuscript), plays a pivotal role by controlling the sparsity and weight distribution based on the physical distance between EEG electrodes. To empirically validate the choice of δ, we conducted a sensitivity analysis by varying its value from 5 to 11.As illustrated in Figure S4, the model performance on both SEED and SEED-IV datasets exhibits a consistent bell-shaped trend, peaking at $\delta = 8$.

This result confirms that $\delta = 8$ achieves the optimal topological balance. Values smaller than 8 result in an overly sparse graph that misses critical local correlations between adjacent electrodes. Conversely,

values larger than 8 significantly increase the weights of distant electrodes, introducing excessive noise and potentially leading to the over-smoothing problem in GCNs. Thus, $\delta = 8$ is selected to ensure the most effective spatial feature aggregation.

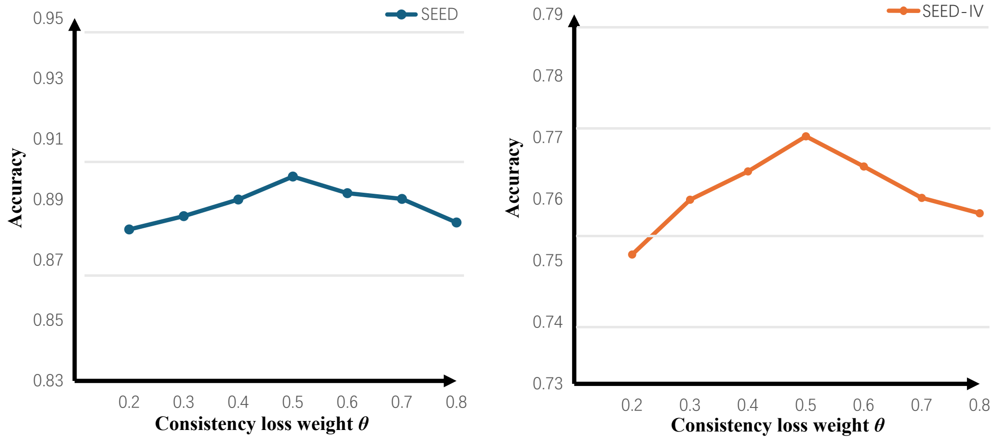

Figure S3. Sensitivity analysis of the multi-view consistency loss weight θ on the SEED (left) and SEED-IV (right) datasets.

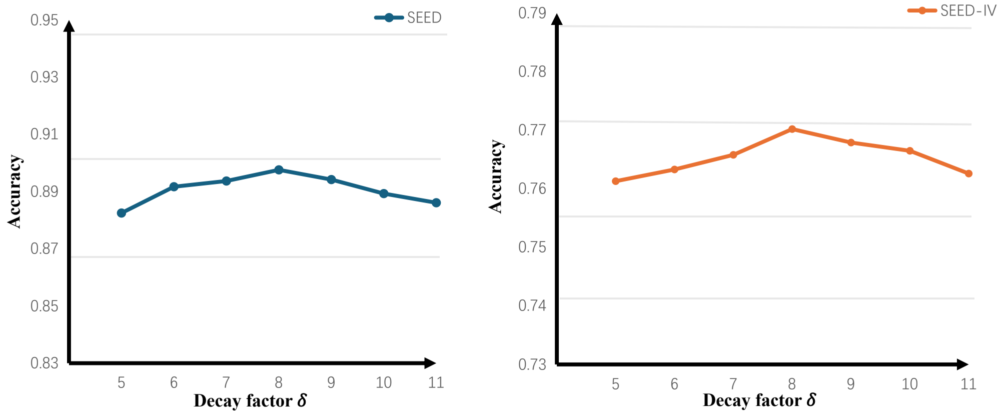

Figure S4. Parameter sensitivity analysis of the decay factor δ on the SEED (left) and SEED-IV (right) datasets.

2 MODULE-LEVEL FEATURE VISUALIZATION

To visually quantify the contribution of the spatial branch to feature discriminability, we employed T-SNE to compare the feature distributions generated by the "w/o Spatial Branch" variant versus the full DSP-MCF model. As shown in Figure S5, the left panel (w/o Spatial Branch) reveals that without spatial topological information, the decision boundaries between emotion categories are ambiguous, specifically showing noticeable overlap between the Light Blue and Dark Blue clusters. In contrast, the right panel (DSP-MCF) demonstrates that integrating the spatial branch significantly sharpens these boundaries. The clusters become more compact, and a clear margin emerges between distinct emotion classes, confirming that spatial topological dependencies are critical for enhancing inter-class separability.

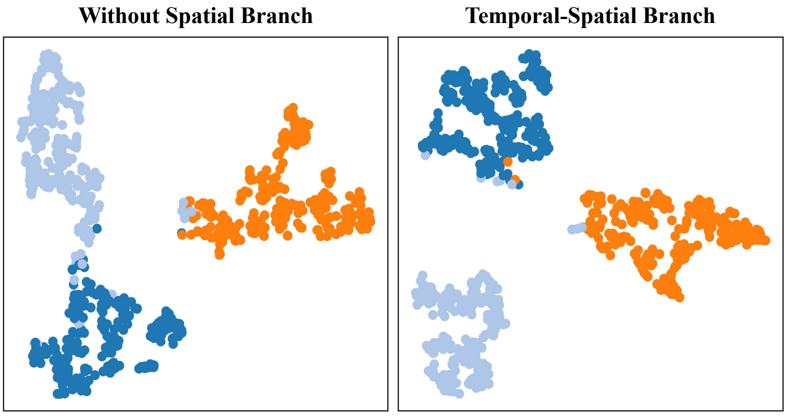

Figure S5. T-SNE visualization of feature distributions on the SEED dataset. The “w/o Spatial Branch” variant shows overlapping clusters, indicating weak separability (left). The full DSP-MCF model produces compact, well-separated clusters (right).

3 PREDICTION DISTRIBUTION ALIGNMENT

To demonstrate the efficacy of the Multi-View Consistency Loss (L_{MVC}) in aligning the semantic representations of incomplete data, we visualized the Kernel Density Estimation (KDE) of prediction probabilities for both the “Original View” and “Masked View”. As shown in Figure S6, the prediction distribution of the Masked View (Red curve) exhibits a high degree of alignment with the Original View (Blue curve), particularly in the high-confidence region (near $x = 1.0$). Despite the input corruption, the modes of both distributions coincide perfectly with no significant distributional shift. This confirms that the symmetric KL-divergence loss successfully forces the model to maintain consistent prediction confidence, effectively mitigating the uncertainty caused by channel loss.

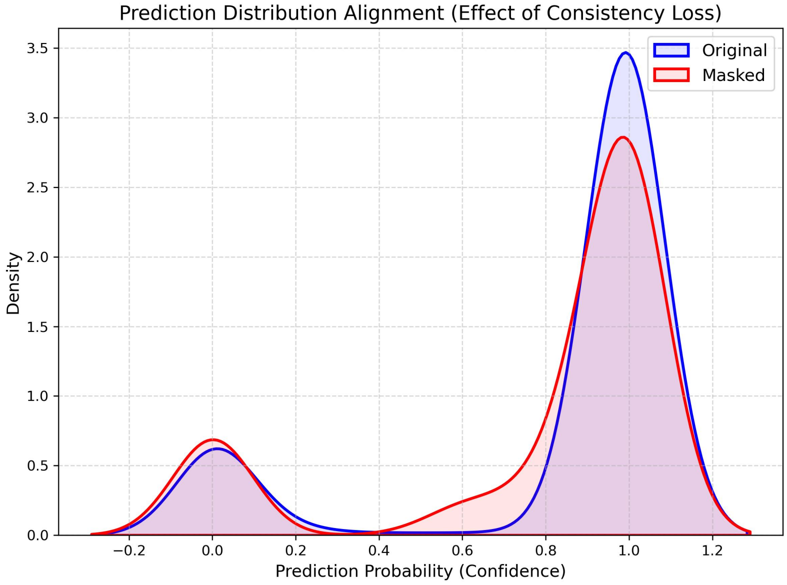

Figure S6. Visualization of prediction distribution alignment using Kernel Density Estimation (KDE). The Red curve (Masked View) closely tracks the Blue curve (Original View), especially in high-confidence regions.
